# Multisensory integration, brain plasticity and optogenetics in visual rehabilitation

**DOI:** 10.3389/fneur.2025.1590305

**Published:** 2025-07-10

**Authors:** Martina Lucchesi, José Fernando Maya-Vetencourt, Dario Rusciano

**Affiliations:** ^1^Department of Biology, Physiology Institute, University of Pisa, Pisa, Italy; ^2^Fidia Pharmaceuticals, Ophthalmology Research, Catania University, Catania, Italy

**Keywords:** multisensory areas, brain plasticity, visual rehabilitation, optogenetics, cross-modality

## Abstract

Multisensory integration enables the brain to integrate information from different sensory modalities while enhancing perception. This principle relies on phenomena of neuroplasticity (i.e., the ability of neuronal networks in the brain to adapt to changing environmental conditions) and is crucial for visual rehabilitation, particularly in hemianopia and retinal degeneration. Here we review emerging experimental approaches and their translational potential for vision recovery in visually impaired patients. Rehabilitation strategies incorporating multisensory training, optogenetics, and pharmacological interventions have demonstrated to be instrumental in restoring visual function by leveraging plasticity of inputs from different sensory modalities. Emerging technologies such as virtual reality and auditory–visual stimulation further optimize neural reorganization. Future research should focus on refining these interventions to enhance sensory compensation and recovery. Understanding the role of multisensory ganglion cells and retinal circuits may unlock new strategies for improving visual function in visually impaired individuals.

## Graphical abstract

Illustration of cutting-edge techniques (i.e., optogenetics, auditory stimulation, visual stimulation, tactile stimulation, pharmacology, virtual reality) that promote brain plasticity and could be employed, alone or in combination, as vision recovery therapies in humans.

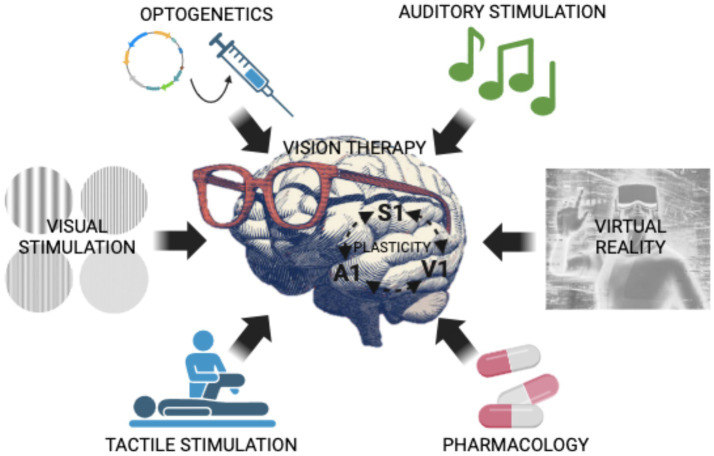

## Introduction

Multisensory integration refers to the brain’s ability to integrate inputs from multiple sensory modalities, creating a cohesive perceptual framework of the world around us. This process is crucial for navigating and interpreting complex environments (1). A large body of evidence highlights that multisensory processing extends beyond primary sensory areas, involving brain regions such as the superior colliculus (SC), pulvinar, and the prefrontal cortex (PFC). Importantly, this integration plays a critical role in spatial perception, allowing the brain to combine sensory cues to construct a more accurate representation of the environment. These findings underscore the complexity and sophistication of the brain’s multisensory processing capabilities. For instance, neurons in the auditory cortex respond to auditory, visual and somatosensory inputs, enhancing spatial and temporal perception (2). In a different study by the same group, it was demonstrated that neural integration of congruent auditory and visual stimuli (meaning cross-modal cues that occur close in space and time), facilitates a cohesive perception of the world (3). This work highlights the SC as a key region where multisensory cells coordinate visual and auditory inputs to optimize responses to complex environments. This coordination is vital for tasks such as locating sounds in space and understanding speech in noisy settings. The reported research has revealed that the brain employs strategies such as causal inference and optimal integration to combine sensory information, thereby enhancing perceptual accuracy (3). The brain uses Bayesian causal inference to determine whether sensory signals should be integrated or segregated, optimizing perception based on the inferred cause of the signals (4). Additionally, Rohe and Noppeney provide evidence that the human brain integrates sensory information in a statistically optimal fashion, weighting cues by their reliability to enhance perceptual accuracy (5). In this review, we shall discuss how advanced strategies generally utilized by the brain to process multisensory information, can be exploited in innovative visual rehabilitation strategies. We will also describe new emerging techniques, which leverage multisensory integration in regions such as the SC or the Pulvinar, to bypass lower damaged structures of the visual pathway. We will also analyze pharmacological treatments potentially able to enhance multisensorial perception aiming at visual rehabilitation. The bibliographic PubMed database was used to identify biomedical literature pertinent to the focus of the review.

### Linking multisensory integration to visual rehabilitation

Cross-modal plasticity is the brain’s ability to reorganize itself after modifications of sensory inputs. It does allow sensory modalities to compensate for the loss of another one, therefore, it can facilitate visual recovery by enhancing the processing of remaining sensory inputs (6). Current rehabilitation techniques leverage multisensory principles to enhance compensatory mechanisms and neuroplasticity-driven recovery, especially in visual rehabilitation strategies.

**Figure 1:**
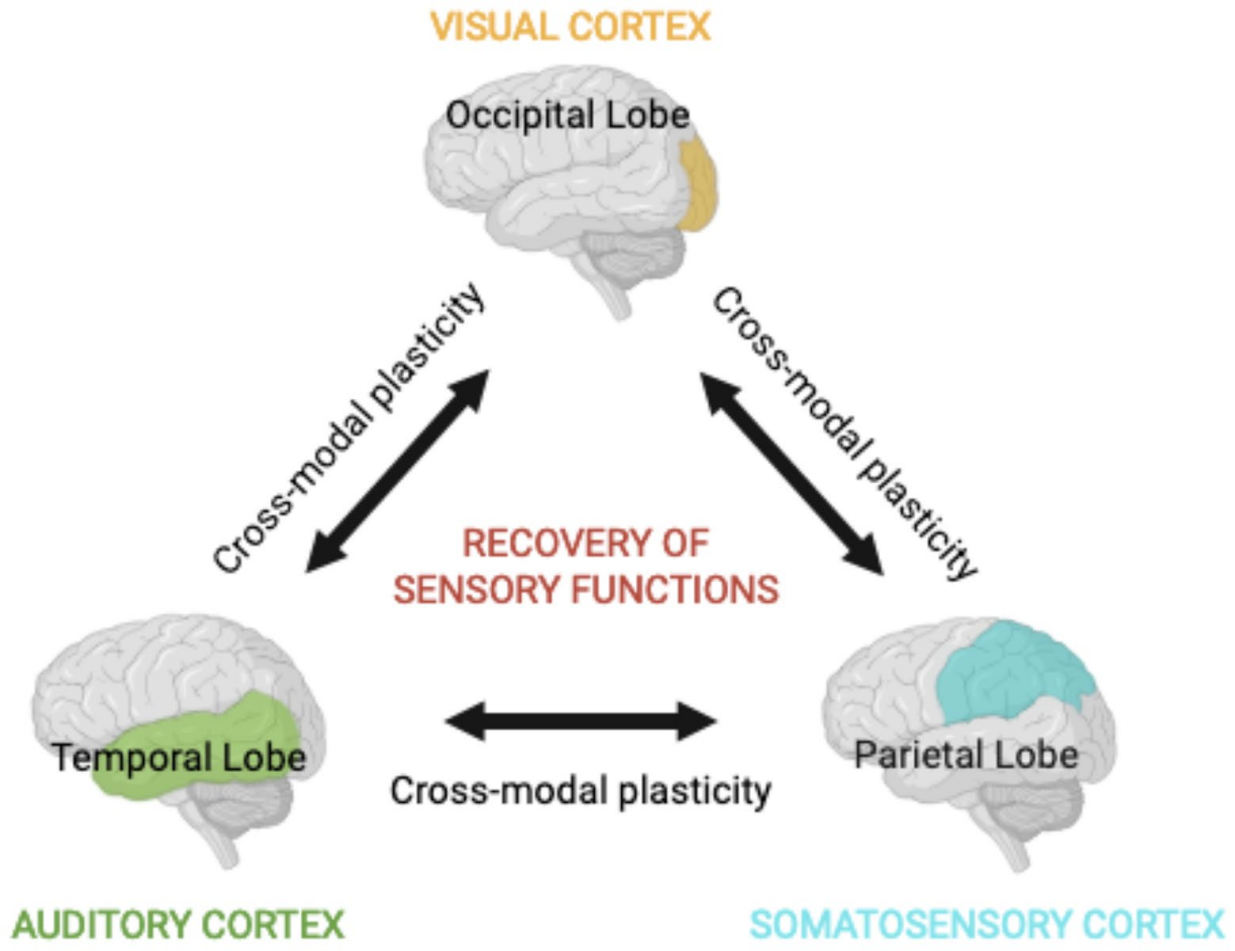
Multisensory integration. The integration of diverse types of information derived from sensory areas, such as visual, auditory, and somatosensory systems, underlies a coherent perception of the environment. The compartmentalized but interconnected nature of the brain ensures that stimulating a single sensory modality has profound effects in the processing of stimuli in other sensory areas. This is possible due to mechanisms of cross-modal plasticity and may facilitate the recovery of sensory functions after sensory deprivation.

These strategies are discussed by Dudon et al. in a seminal publication focusing on individuals with homonymous visual field defects resulting from post-chiasmatic brain injuries. These visual rehabilitation techniques, falling under paradigms of compensation and restoration, focus on improving patients’ visual performance and quality of life. The authors categorize visual rehabilitation into three main approaches: Visual Scanning Training (VST), Audio-Visual Scanning Training (AViST), and Vision Restoration Training (VRT). VST enhances compensatory eye movements by training patients to scan their visual field systematically, thereby mitigating the functional impacts of scotomas. AViST integrates multisensory stimulation, combining visual and auditory inputs to engage the SC and related neural circuits, improving spatial awareness and oculomotor functions. Both VST and AViST focus on compensation by utilizing intact neural pathways. Conversely, VRT aims to restore lost vision by stimulating residual visual field areas, leveraging neuroplasticity to enhance the activity of spared neural tissue. The authors emphasize the importance of regions with residual vision and their potential to contribute to functional improvements through targeted repetitive stimulation. This approach challenges the traditional view that post-acute vision loss is permanent. The study also highlights the emerging role of multisensory integration and neuroplastic phenomena in enhancing visual rehabilitation outcomes (Figure 1). AViST, for instance, demonstrates the capacity of combined sensory stimuli to improve visual detection in the blind field, suggesting that recruiting spared pathways can lead to functional compensation. Dundon and colleagues underscore that while these approaches are distinct, they are not mutually exclusive, with evidence indicating overlap in their neural mechanisms. They advocate for further exploration into the neural underpinnings of these methods to optimize treatment strategies and highlight the need for combining compensatory and restorative techniques to achieve comprehensive visual rehabilitation (7).

### The role of multisensory plasticity in enhancing visual rehabilitation outcomes

Diverse studies show that short-term monocular deprivation enhances auditory and tactile responsiveness, revealing the potential for leveraging multisensory interactions in visual rehabilitation. Retinal plasticity also plays a role, as damage-induced remodeling facilitates functional recovery, opening avenues for therapies that utilize existing neural capabilities. Several years of research have established the respective roles of multisensory integration, optogenetics and pharmacological strategies, in visual rehabilitation.

#### Cross-modal plasticity in vision

Plasticity enables the brain to adapt to changing environmental conditions, a phenomenon well-exemplified by cross-modal reorganization. Federici et al. (8) demonstrated that short-term monocular deprivation enhances the responsiveness of non-visual sensory modalities, such as auditory and tactile systems, in regions associated with visual processing. This adaptability provides a neural framework for leveraging auditory and tactile stimuli in visual rehabilitation. The retina itself exhibits plasticity, as shown by Strettoi et al. (9), who highlighted how damage-induced remodeling of retinal circuits facilitates functional recovery. Such intrinsic plasticity, which includes the influence from other brain areas, offers novel avenues for developing therapies that utilize existing neural capabilities to restore vision.

#### Role of multisensory cells in visual rehabilitation

Multisensory cells in the SC, which integrate inputs from multiple modalities, are pivotal for rehabilitation strategies using auditory and tactile cues to compensate for visual loss. Their function is closely tied to neuroplasticity and cross-modal adaptation, as they contribute to the brain’s ability to reorganize and compensate for sensory deficits. These cells help direct attention to relevant stimuli, a mechanism that forms the foundation of many multisensory training techniques. Evans showed how selective attention modulates cross-modal interactions, allowing these cells to prioritize relevant stimuli for rehabilitation purposes. Such mechanisms are foundational for therapies targeting attention-based tasks in visually impaired patients (10). Okada et al. (11) conducted a functional magnetic resonance imaging (fMRI) study revealing that the auditory cortex enhances audiovisual speech perception, underscoring the potential of multisensory training to reengage brain regions associated with vision through compensatory sensory pathways.

#### Technological advances in multisensory integration

Cornelio et al. (12) reviewed emerging technologies that enhance multisensory integration research. Volumetric displays, virtual reality, and tactile feedback systems have transformed rehabilitation by enabling immersive environments where patients can practice real-world scenarios. These technologies combine visual, auditory, and tactile feedback to promote neuronal plasticity and improve spatial navigation skills. Virtual reality simulations paired with auditory cues have been particularly effective in enhancing depth perception and environmental awareness. For instance, multisensory setups that pair visual and tactile stimuli can retrain neural circuits responsible for spatial orientation (12).

#### Mechanisms of multisensory perception

The McGurk effect is a well-known phenomenon in the field of multisensory integration research. It illustrates how our brain combines conflicting auditory and visual information to create a unified perceptual experience. When the auditory component of a speech sound is paired with a mismatched visual component, such as seeing someone say “ga” while hearing “ba,” the brain often fuses these inputs, leading to the perception of a different syllable, like “da.” This effect highlights the brain’s reliance on integrated sensory processing to make sense of the world. Research by Okada et al. and Bizley and King delved into the neural mechanisms underlying the McGurk effect. They found that this perceptual fusion occurs because the brain prioritizes coherent sensory experiences, even when the inputs are conflicting. This interplay between senses demonstrates the depth of sensory integration and the brain’s ability to adapt and create a consistent perception from disparate sources (2, 11). This phenomenon has significant implications for rehabilitation strategies, as it demonstrates the brain’s ability to integrate sensory inputs to enhance perception. Training programs that leverage audiovisual integration, particularly in speech and spatial awareness rehabilitation, can help individuals with sensory deficits develop compensatory mechanisms that improve functional outcomes. Building on these foundational studies, Rowland et al. applied the principles of cross-modal integration to clinical populations. They developed training programs that use multisensory stimuli to enhance functional vision in individuals with visual impairments. By combining auditory and visual cues in a structured training regimen, they achieved measurable improvements in patients’ visual processing abilities. This approach leverages the brain’s plasticity and its capacity to reorganize and strengthen neural pathways through multisensory stimulation (13). Overall, these studies underscore the importance of multisensory integration in both basic perceptual processes and applied clinical interventions. The McGurk effect serves as a powerful example of how our brains synthesize information from different senses to create a coherent understanding of our environment, and how this principle can be harnessed to improve sensory function in therapeutic settings.

### Optogenetics and multisensory integration: a new frontier in visual restoration

In addition to virtual reality technologies, optogenetics may also be instrumental in promoting cross-modal plasticity. Optogenetics is an innovative and powerful approach that modifies neuronal activity using light-sensitive proteins, combining genetic engineering and light stimulation (14, 15). It does allow researchers to manipulate the timing and pattern of neuronal activity in specific neural subtypes with milliseconds precision (16–19). This revolutionary tool has provided unparalleled insights into the understanding of mechanisms that underlie sensory perception (20, 21).

#### Optogenetically induced remodeling of neural circuitries

Leong et al. (22) used a combination of fMRI and optogenetics to explore how primary visual cortex (V1) inputs cross-modally affect auditory processing in the midbrain of rats. The optogenetic activation of excitatory pyramidal neurons in the infragranular layers of V1 significantly enhanced sound-evoked responses in the inferior colliculus, demonstrating that V1 inputs facilitate sound processing (22). The effect of auditory inputs on visual perception is also a hot spot in current neuroscience research. It has been demonstrated that activation of the primary auditory cortex (A1) by a noise drives local GABAergic inhibition on supragranular pyramids of V1, this effect being dependent on cortico-cortical connections (23). This phenomenon seems to be generated by sound-driven excitation of infragranular visual cortical neurons, which was confirmed by the fact that light pulses in the V1 of mice expressing the photosensitive protein channelrhodopsin-2 (ChR2) in layer 5 pyramids, hyperpolarized responses in layer 2/3 pyramid cells (23). Consequently, visually driven synaptic responses were reduced upon bimodal stimulation. These findings suggest that A1 activation by salient stimuli reduces potentially distracting signals in V1, by recruiting inhibitory circuits. There is also evidence that prolonged presentation of auditory stimuli recalibrates the orientation selectivity of V1 neurons in cats. The extracellular recording of neuronal activity in anesthetized animals revealed that orientation tuning curves in both supragranular and infragranular neurons of V1 markedly change after long-term presentation of acoustic stimuli (24). Accordingly, pure tones improve the neural representation of the orientation and direction of visual stimuli in mice (25). All these findings demonstrate that V1 pyramidal neurons dynamically integrate features of sound. Consistently, orientation selectivity in superficial layers of V1 in mice is sharpened in the presence of a sound or after the optogenetic activation of A1 areas (26).

The role of multisensory integration in the cross-modal interaction between vision and touch has also been investigated. Olcese et al. (27) explored this issue in a bimodal brain region, named the rostrolateral area (RL), that is located between the rostral part of V1 and the caudal part of the primary somatosensory cortex (S1). Many neurons in this visuo-tactile region are unimodal but they receive subthreshold inputs from other sensory modalities. The response of pyramidal cells in layer 2/3 of the RL to bimodal stimuli (visual + tactile) was found to be higher as compared to the strongest unimodal stimulus. The authors also demonstrated that the integration of sensory inputs is orchestrated by layer-specific mechanisms. This was shown by optogenetically activating Pv^+^ GABAergic neurons while recording electrophysiological responses from pyramidal cells. Interestingly, light stimulation increased Pv^+^ GABA cell activity to a greater extent when bimodal sensory stimuli were presented as compared to unisensory trials. Consequently, Pv^+^ interneuron photoactivation with multisensory trials resulted in larger reductions of synaptic responses in pyramidal cells as compared to unisensory stimuli (27). This highlights the existence of a relationship linking multisensory properties of excitatory and inhibitory cell types in cortical microcircuits. It might be interesting to develop an optogenetic approach to obtain a reduction of Pv^+^ inhibitory cells, rather than an enhancement, to increase the firing activity of pyramidal cells. This may be of relevance for visually impaired patients as one could, in principle, bypass the damaged retina and directly stimulate associative areas that might have a beneficial impact on visual perception. Taken together, these findings suggest that integrating optogenetic approaches with multisensory training could enhance functional recovery in visually impaired individuals.

### Multisensory rehabilitation strategies

From a practical point of view, different multisensory rehabilitation approaches have been proposed.

1. *Hemianopia and multisensory training:* In their 2023 study, Rowland et al. (13) explored a novel rehabilitation technique for hemianopia, a condition characterized by blindness in half of the visual field, often resulting from stroke or brain injury. The researchers combined auditory and visual stimuli to create a multisensory training program. This approach leverages the brain’s natural ability to integrate information from different senses, enhancing neural plasticity and recovery. The study involved exposing patients to congruent (matching in time and space) auditory and visual stimuli within their blind visual field. Over several weeks of training, participants showed significant improvements in their ability to detect and describe visual stimuli in the previously blind areas. This improvement was attributed to the synergistic effect of using both auditory and visual inputs, which helped to re-engage and strengthen the neural pathways involved in visual processing. The results of this study are promising, suggesting that multisensory training could be a rapid and effective method for restoring visual function in individuals with hemianopia (13).
2. *Cross-modal correspondences in visual processing*: In his 2020 research, Spence (28) delved into the intriguing phenomenon of cross-modal correspondences, which refers to the natural associations our brains make between different sensory modalities. Specifically, Spence investigated how pairing temperature cues with visual stimuli can enhance neural responses, a concept with significant implications for rehabilitation strategies. Spence’s study focused on the idea that our sensory systems are interconnected, and that stimuli from one sense can influence the perception and processing of stimuli from another. For instance, people often associate certain colors with specific temperatures (e.g., red with warmth and blue with coolness). By leveraging these natural associations, Spence demonstrated that presenting congruent temperature and visual cues together can amplify neural activity and improve sensory processing. This research opens new avenues for rehabilitation, particularly for individuals recovering from sensory impairments. By integrating temperature cues with visual stimuli, therapists can create more effective multisensory training programs. These programs can help rewire the brain and enhance recovery by taking advantage of the brain’s ability to form and strengthen connections between different sensory modalities. Overall, Spence (28) work highlights the potential of using novel sensory combinations to improve rehabilitation outcomes, offering a promising approach for enhancing neural plasticity and sensory integration.
3. *Long-term sensory stimulation after stroke*: In their 2016 study, Tinga et al. (29) investigated the impact of sustained multisensory stimulation on stroke recovery, particularly focusing on visual rehabilitation. The researchers emphasized the importance of long-term stimulation protocols that engage multiple sensory systems—such as visual, auditory, and somatosensory stimuli—to enhance recovery outcomes. The study highlighted that sustained multisensory stimulation can significantly improve both low-level perceptual deficits (like visual field defects) and higher-level cognitive sensory deficits (such as visual attention and processing) in stroke patients. By continuously stimulating different sensory modalities over an extended period, brain’s plasticity is harnessed, promoting the reorganization and strengthening of neural pathways involved in sensory processing. Tinga et al. (29) provided valuable insights into designing effective long-term visual rehabilitation strategies. They suggested that incorporating multisensory elements into rehabilitation programs can lead to more robust and enduring improvements in visual function. This approach not only aids in the recovery of lost sensory functions but also enhances the overall quality of life for stroke survivors. The findings from this study underscore the potential of long-term multisensory stimulation as a powerful tool in stroke rehabilitation, offering a promising direction for future research and clinical practice (29).
4. *Audio-visual stimulation of the damaged retina*: In her 2014 study, Zelinsky (30) explored the role of auditory and visual integration in enhancing recovery for individuals with brain injuries. This research focused on how combining multisensory stimuli can help retrain damaged neural circuits and improve overall neural responsiveness, providing practical applications for post-trauma therapies. Zelinsky (30) approach involves using both auditory and visual inputs to stimulate the retina, particularly the peripheral retina, which is often overlooked in traditional treatments. By activating these peripheral pathways, the brain can receive enhanced sensory input, which aids in the reorganization and strengthening of neural connections. This method leverages the brain’s inherent plasticity, allowing it to adapt and recover more effectively from injury. The study demonstrated that integrating auditory cues with visual stimuli can significantly improve sensory processing and neural responsiveness. This multisensory integration helps to re-engage and retrain the damaged circuits, leading to better functional outcomes for patients. For instance, patients who underwent this type of therapy showed improvements in their ability to perceive and respond to visual stimuli, which is crucial for daily activities and overall quality of life. Zelinsky (30) findings highlight the potential of using multisensory stimulation as a powerful tool in brain injury rehabilitation. By incorporating auditory and visual integration into therapeutic protocols, clinicians can design more effective and comprehensive rehabilitation strategies that address the complex needs of brain injury patients.
5. *An environment rich in sensory stimulation and amblyopia recovery*: Living in an environment rich in sensory stimuli, a condition known as environmental enrichment (EE), markedly shapes brain structure and function. An enriched environment is a multisensory approach whereby enhanced stimulation is provided at multiple levels: sensory, social, motor, and cognitive (31). In animals, it consists of wide cages where they are reared in large social groups and in the presence of a variety of stimulating objects (e.g., toys, stairs, tunnels, nest materials, running wheels) that are regularly changed and substituted with others to stimulate explorative behavior, curiosity, attentional processes, and physical activity. This behavioral approach not only accelerates V1 development (32) but has also been used to enhance plasticity in the adult V1 (33–35). This outlines the therapeutic potential of this strategy in pathological conditions where plasticity is compromised. Amblyopia, for instance, is a pathology in which vision in one eye is markedly impaired due to an abnormal visual experience during development (36). This pathology can be effectively treated by eye occlusion or penalization in early life (37) but it is difficult to treat in adulthood due to a decrease of brain plasticity that occurs with age (38–41). Not surprisingly, EE promotes the recovery of normal visual functions, both electrophysiologically and behaviorally, in adult amblyopic animals (42). This phenomenon seems to be due to a reduction of intracortical inhibition, which parallels an increased expression of the neurotrophic factor brain-derived neurotrophic factor (BDNF). The advantage of this environmental approach, in addition to its non-invasiveness and ease of application, lies in its already proven translatability to humans. Indeed, it has been previously reported that voluntary levels of exercise (an important component of EE) potently boost plasticity in the adult human visual system (43–45).

### Optogenetics in combination with other visual rehabilitation techniques

A promising direction for visual rehabilitation relays on the use of optogenetics in higher-order regions of the visual pathway with multisensory integration capabilities. By leveraging the brain’s ability to integrate sensory cues, targeted optogenetic interventions could enhance neural reorganization and optimize functional recovery.

It has been reported that optogenetic activation of the visual thalamus, i.e., the lateral geniculate nucleus (LGN), generates artificial visual percepts in the absence of real visual input (46). Neurons of the LGN were modified in tree shrews to express the light-sensitive protein ChR2 and were later activated with light pulses delivered via an optic fiber. Remarkably, when the LGN was activated, the animals behaved as if they were responding to real visual stimuli, even though no actual visual input was present (46). These results suggest that LGN optogenetic activation may be sufficient to produce an artificial experience of vision while bypassing the impaired retina. A similar study involving the SC was also conducted. In a mouse model of glaucoma induced by laser photocoagulation, the stabilized step function opsin (SSFO) light-sensitive channel was expressed in the SC and light stimuli were used to activate SC neurons. Notably, despite their damaged retina, animals were able to respond to optogenetic stimuli, as evidenced by behavioral tests (47). Therefore, optogenetic LGN or SC stimulation in combination with cross-modal cutting-edge technologies might provide novel interesting strategies for restoring vision-like perception in patients affected by visual impairments, where retinal cells may be damaged but the LNG, SC, and V1 are still intact.

Different works used optogenetic tools to explore the interaction of SC and V1 in healthy sighted animals, such research having profound implications for visual rehabilitation strategies development. In a 2018 study, Ahmadlou et al. expressed the protein ChR2 in mouse SC neurons. V1 responses to the artificial light stimuli in the SC were recorded when animals were engaged in visual tasks requiring attention (eye movements). The authors report that superficial layers of the SC can either increase or inhibit the responsiveness of V1 neurons, depending on the task and the type of stimulus. During these tasks, SC activation enhances V1 responsiveness but if the visual stimulus was irrelevant, SC activation results in suppression of V1 responses. This suggests that the SC in concert with other brain areas (48–50) contributes to the ability to focus attention and enhance relevant visual processing while reducing interference from irrelevant information (51). In visual rehabilitation strategies, enhancing the attention-focusing ability of the SC via optogenetic techniques, might, in principle, improve the ability of visually impaired patients to interact with the environment and navigate it more effectively.

**Figure 2:**
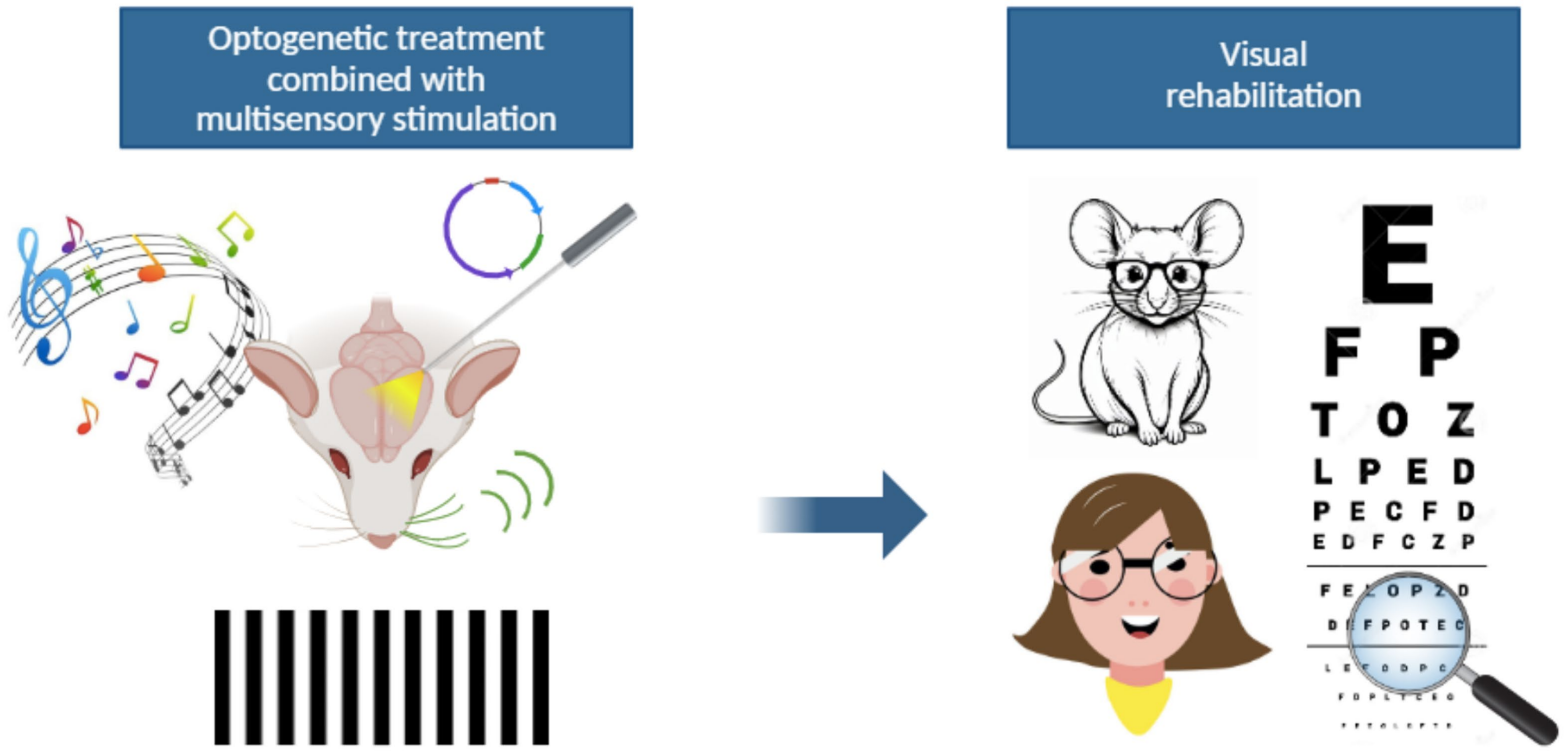
A possible new strategy for visual rehabilitation. The integration of optogenetic approaches with multisensory stimulation might represent a novel visual rehabilitation strategy for patients with visual impairments. The optogenetic stimulation of multisensory associative areas could be an indirect way to activate the visual pathway bypassing the damaged retina. At the same time, auditory or tactile stimuli may provide a way to increase visual perception. The ability of the brain to integrate information coming from diverse sensory areas might therefore lead to improved visual outcomes thanks to phenomena of cross-modal plasticity.

Another important region for sensory perception is the prefrontal cortex (PFC), which is specifically involved in top-down control of attentional mechanisms, meaning it is crucial to select and sustain attention on important stimuli while filtering out distractions (52, 53). PFC interacts with the SC to control voluntary eye movements and regulate attentional processes. It modulates SC activity based on cognitive goals to guide eye movements toward relevant targets (54, 55). The SC, in turn, provides sensory information and responses that the PFC uses for more complex decision-making (54). The pulvinar, a key nucleus of the thalamus, located in its posterior region, is another fundamental area for visual attention. It acts as a hub for sensory information processing, filtering distractions via amplifying relevant signals and suppressing irrelevant ones while coordinating eye movements (56). This structure is deeply involved in both bottom-up and top-down attention processes and, therefore, interacts with both the PFC and the SC to enhance attention toward a chosen target, allowing sensory-motor coordination (57–60). The pulvinar elaborates and transmits visual information from the SC to higher-order areas like the PFC and parietal cortex. This enables cognitive control over attention shifts (61, 62). Interestingly, the optogenetic activation of the frontal cingulate cortex (Cg) in the brain of mice, enhances V1 neural responses and improves visual discrimination (63). It has also been reported that the optogenetic manipulation of the Cg modulates visually guided behaviors, this effect being mediated by collateral projections to both the motor-related layers of the SC and the lateral posterior nucleus of the thalamus (LP) (64). All these findings suggest that the optogenetic modulation of PFC, SC, and Pulvinar, in parallel to current visual rehabilitation strategies may also be a successful approach for vision restoration in the blind (Figure 2). The application of optogenetics in multisensory regions has significantly expanded our understanding of visual processing. Future research is likely to focus on refining engineered light sensors, improving delivery mechanisms, and developing more selective tools for manipulating specific neuronal circuits, in order to exploit the multisensory integration ability of structures such as SC, PFC, and Pulvinar.

It is interesting to note that all findings above mentioned are in line with recent observations on the therapeutic effect of optogenetic stimulation of retinal ganglion cells (RGCs) in animal models and patients afflicted by neurodegenerative blindness. Innovative approaches that partially restore vision by means of optogenetics are based on targeting RGCs with photosensitive proteins. A recent *in vivo* imaging study employed this technique in a non-human primate model of photoreceptor degeneration and successfully reported RGC activity more than 1 year after receiving the genetic therapy, thus suggesting a long-lasting effect on inner layers retinal stimulation (65). More recently, the first study performed in humans was conducted on a 58-year-old male individual who had been blind for 20 years because of retinal neurodegeneration. The patient received an intraocular injection of an AAV vector encoding the optogenetic sensor (Chrimson R) targeting the RGCs. This treatment, followed by 7 months of training with complex exercises plus light stimulation via engineered goggles, partially restored the visual capabilities of the patient (66). This work is part of an ongoing clinical trial (NCT03326336) and represents the first reported case of functional recovery in patients with neurodegenerative blindness by means of optogenetics.

### Limitations and risks on the use of optogenetics to improve visual therapies

The use of cutting-edge technologies as therapeutic strategies that leverage the brain’s ability to integrate modifications of neuronal circuitries, is highly challenging. Although groundbreaking, most of the studies using optogenetics to modulate neuronal circuitries rely heavily on animal models. To make neurons sensitive to light, it is necessary to introduce genes that encode light sensitive proteins such as channelrhodopsin via viral vectors or other genetic engineering techniques. Gene therapies carry significant clinical risks, such as immune response against viral vectors, and pose ethical and regulatory issues especially for applications in the human brain. Regulatory authorities also require very rigorous evidence before approving brain gene therapies.

One important issue regarding the application of optogenetics in clinics is the penetration of light into the neural tissue. Visible light, which is necessary for enhancing or inhibiting neuronal excitability in a light-dependent manner, does not penetrate deep into the brain tissue. This is a significant limit as most light sensitive proteins require short wavelengths (~blue to green) to be activated and consequently modulate the cell’s membrane potential. Hence global efforts have been made in the development of photo sensitive proteins sensible to long wavelengths (~red to infrared), which can penetrate more into the neural tissue. Nowadays, most studies performed in animal models are conducted using implanted optical fibers after viral infections. This experimental approach, unfortunately, can be barely foreseen in human applications. Implanting strategies into the brain is invasive and carries risks of undesired infection, inflammation, and tissue damage. Given the pace of technological advances, however, this challenge is likely to be surpassed in the years to come.

The surgical implant of optical devices to stimulate neurons with external light sources emphasizes the need for miniaturized neuronal interfaces (i.e., small devices to achieve light stimulation). Clinical issues associated with this practice include neurosurgical risks, difficulties in long-term maintenance, timelines of therapies, and biocompatibility of the materials. The later consideration is particularly important to ensure getting a low inflammatory response to the stimulation device that is implanted in the brain. All these considerations highlight that optogenetics, although extremely successful in animal models, may be much more difficult to apply in a safe and controlled way in humans. One should also consider differences in the dimension and complexity of the human brain, as compared to that of other species, as well as ethical and regulatory aspects of genetic brain modification. Moreover, light-sensitive protein expression may vary over time. Therefore, responses to light might decline and require re-treatment or modifications over time. This shows that timelines of potential treatments must be carefully studied and considered before clinical application. All these findings indicate that there are still several issues that need to be addressed before employing optogenetics in combination with other emerging technologies as a visual therapy in humans.

### Pharmacological approach to multisensorial visual rehabilitation techniques

Enhancing multisensorial perception for visual rehabilitation using pharmacological agents is also a growing area of interest in neuroscience. Combining these approaches with training paradigms such as audio-visual integration or tactile-visual substitution could accelerate recovery by fostering neural adaptability. While the direct application of drugs to potentiate multisensory integration is not yet a widespread clinical practice, experimental evidence suggests several pathways and pharmacological agents that might contribute to this goal indirectly. Different strategies, as supported by feasible scientific evidence, although not yet clinically validated, are:

1. *Modulating neuroplasticity*: Drugs that target neuroplasticity mechanisms, like N-methyl-D-aspartate (NMDA) receptor modulators, hold great promise for enhancing multisensory integration. NMDA receptors are a type of glutamate receptor that play a critical role in synaptic plasticity. Neuronal plasticity is essential for learning, memory, and overall brain function. Modulating these receptors can significantly impact neuroplasticity and, consequently, cognitive processes and recovery from brain injuries. D-cycloserine is a notable example. It is a partial agonist of the NMDA receptors, meaning it binds to the receptor and activates it, but not to its full potential. This partial activation increases synaptic plasticity without causing excessive stimulation, which can be harmful. Additionally, D-cycloserine binds to the glycine site on the NMDA receptor, facilitating the receptor’s activation. This enhances long-term potentiation and long-term depression, which are processes that strengthen and weaken synapses, respectively (67). By enhancing synaptic plasticity, D-cycloserine can improve learning and memory processes. The modulation of NMDA receptors by D-cycloserine can support rehabilitation efforts in conditions like dementia or after brain injuries by promoting the reorganization and repair of neural networks (enhancement of neuronal plasticity), therefore, leading to improved functional outcomes (67). The drug may also improve cognitive deficits by enhancing synaptic plasticity and connectivity, in which case, in patients recovering from stroke or traumatic brain injury, it can aid in the rehabilitation process by promoting neural repair and functional recovery (67). Drugs like D-cycloserine have been demonstrated to enhance synaptic plasticity, supporting multisensory learning processes and rehabilitation after brain injury (68–70). Non-pharmacological methods to enhance neuroplasticity have also been proposed, which can complement pharmacological approaches (71).

Similarly, selective serotonin reuptake inhibitors (SSRIs) like fluoxetine are known to increase levels of BDNF. BDNF is crucial for synaptic repair and plasticity, which are essential for maintaining and improving brain function (72, 73). Research has shown that SSRIs increase neuronal plasticity by decreasing the ratio of inhibition/excitation (74–76). This is thought to cause a downstream enhancement of BDNF levels, which in turn promotes further plasticity and synaptic repair. This is particularly important in the context of depression, where BDNF levels are often reduced (77). By increasing BDNF, SSRIs help to restore normal synaptic function and improve mood and cognitive function. BDNF plays a pivotal role in supporting synaptic plasticity, employing several mechanisms to enhance the brain’s ability to adapt and learn. One key function of BDNF is its ability to enhance synaptic transmission by increasing the efficiency of communication between neurons, promoting the release of neurotransmitters, and boosting the responsiveness of postsynaptic cells. This ensures that signals are transmitted more effectively, which is essential for neural communication (78). Additionally, BDNF promotes the growth of synapses, both by stimulating the formation of new connections and by strengthening existing ones. This synaptic remodeling is fundamental to processes such as learning and memory, enabling the brain to adapt to new information and experiences (79). Moreover, BDNF provides neuroprotective benefits, shielding neurons from potential damage and supporting their survival. This neuroprotective role not only ensures the maintenance of healthy neural networks but also contributes to the brain’s resilience in the face of injury or degenerative processes (80). Through these mechanisms -enhancing synaptic transmission, promoting synaptic growth, and providing neuroprotection- BDNF serves as a critical factor in maintaining and optimizing the brain’s plasticity and overall functionality.

2. *Enhancing cortical excitability*: The use of drugs able to enhance cortical excitability can lead to improvements in attentional processes and overall sensory responsiveness. Modafinil and atomoxetine are well-representative examples of this category. Modafinil increases levels of catecholamines, serotonin, glutamate, orexin, and histamine in the brain, which can enhance attention and executive functions (81, 82). Atomoxetine works by inhibiting the reuptake of norepinephrine, which helps improve attention and reduce impulsivity (81, 83, 84). Cholinergic enhancers like donepezil have also shown potential in boosting sensory discrimination and perceptual learning (85, 86). Donepezil is a cholinesterase inhibitor that increases the levels of acetylcholine in the brain, a neurotransmitter crucial for various cognitive functions. Research has demonstrated that donepezil can enhance perceptual learning, which is the improvement in sensory task performance through training. Indeed, donepezil administration during perceptual tasks increases the magnitude of learning and its specificity to the trained stimuli (85, 87). This means that individuals taking donepezil can experience more pronounced and long-lasting improvements in tasks such as motion direction discrimination (87). Moreover, the benefits of cholinergic enhancement with donepezil are not just acute but persist for several months after the training period (87). This long-term effect suggests that donepezil could be particularly useful in clinical settings for enhancing cognitive training outcomes and potentially aiding in the rehabilitation of sensory and cognitive functions. Caution, however, must be taken as an excessive increase in cortical excitability might, potentially, induce epileptic seizures (88–90).
3. *Promoting sensory plasticity in visual and multisensory cortices*: Another neurotransmitter that regulates synaptic plasticity is Dopamine, which is crucial for various physiological functions, including motor control, emotional regulation, and reward mechanisms. Different studies have shown that dopamine can modulate synaptic plasticity. In line with this, dopaminergic agents, including L-DOPA, have been noted for their ability to enhance reward-related plasticity and sensory learning, especially in tasks requiring perceptual adaptation (91). As previously mentioned, the GABAergic modulation plays a significant role in neural plasticity. GABA is the primary inhibitory neurotransmitter in the brain, and its modulation can influence the balance between excitation and inhibition in neural circuits. Selective inhibitors of GABA receptors can reduce inhibition in sensory cortices, which may enhance plasticity and integration. This reduction in inhibition allows for greater synaptic changes and can facilitate learning and adaptation in sensory processing. This mechanism is particularly important in contexts where increased plasticity is beneficial, such as in rehabilitation following neural injury. Therefore, the GABAergic modulation, through selective inhibitors using appropriate non-epileptic doses, may temporarily reduce inhibition in sensory cortices, thereby fostering plasticity and integration (92, 93).
4. *Multisensory pathway modulation*: Psychoactive compounds targeting serotonin receptors, such as psilocybin, have demonstrated the ability to enhance sensory perception and multisensory integration under controlled conditions (94). When ingested, psilocybin is converted into psilocin, which interacts primarily with the serotonin 2A receptors in the brain. This interaction leads to a cascade of neurochemical events that can alter perception, mood, and cognition (95). Under controlled conditions, such as in clinical trials or therapeutic settings, psilocybin has been shown to enhance sensory perception, making colors appear more vivid, sounds more distinct, and even inducing synesthesia, where senses overlap (e.g., seeing sounds or tasting colors) (96). These effects are believed to be due to increased connectivity and communication between different brain regions, a phenomenon often referred to as brain hyperconnectivity (97). Moreover, psilocybin can enhance multisensory integration meaning that it facilitates a more profound and integrated processing of sensory and emotional experiences (95).

Additionally, intranasal oxytocin (IN-OT) has been studied extensively for its effects on social cognition and perceptual processes (98). IN-OT has been shown to enhance social perception and interactions. This includes improved emotion recognition, increased trust, and better interpretation of social cues. These effects are thought to be due to oxytocin’s role in modulating neural circuits involved in social behavior (99, 100). Moreover, IN-OT can influence multisensory integration, particularly in how the brain processes and combines information from different sensory modalities. For instance, it has been found to enhance the integration of visual and tactile stimuli, which is crucial for tasks like object recognition (101). These findings suggest that IN-OT can improve both social and perceptual domains by enhancing the brain’s ability to integrate inputs from different sensory systems, leading to better social interactions and perceptual accuracy.

In the context of visual rehabilitation, combining pharmacological agents with multisensory training paradigms, such as audio-visual integration or tactile-visual substitution, could accelerate recovery by fostering neuroplasticity. For instance, D-cycloserine paired with auditory–visual training protocols could optimize cortical rewiring for improved sensory integration (70). Furthermore, interventions aiming to restore plasticity using fluoxetine or environmental enrichment strategies hold promise for rehabilitative settings (31, 33, 38, 45, 75, 102). Along this line, also the use of melatonin could provide some benefits for neuronal enhancement (103). Melatonin plays a role in neuronal plasticity, enhancing synaptic transmission, promoting the formation and strengthening of synapses, and finally improving the brain’s ability to adapt and reorganize itself (104, 105). Moreover, melatonin has been shown to support the growth of new neurons, particularly in the hippocampus, a region involved in memory and learning (106). Enhanced neurogenesis can lead to better cognitive functions, including sensory perception. This can improve the brain’s ability to process and integrate sensory information from different sources. However, while these mechanisms suggest that melatonin could enhance sensory processing and integration, more research is needed to fully understand its potential in this area.

### Future perspectives

In the context of visual rehabilitation, we hypothesized that multisensory integration may facilitate neuronal plasticity, favoring functional recovery in visually impaired patients. Multisensory training, based on the presentation of congruent audiovisual stimuli, has been shown to improve visual reactivity through the stimulation of neuronal circuits. In this context, optogenetic tools represent a promising breakthrough in vision rehabilitation strategies. Once surviving retinal neurons are irreversibly damaged, optogenetics offers the possibility to bypass them, by acting on higher subcortical and/or cortical areas. Moreover, optogenetic tools could help dissect neural circuits involved in multisensory integration, identifying key nodes and interactions that can be targeted by vision rehabilitation therapies. Optogenetic stimulation could also be combined with structured multisensory rehabilitation training designed for visual re-establishment to enhance neural reorganization and functional recovery in visually impaired patients.

## Conclusion

Multisensory integration is a fundamental mechanism that underpins sensory perception, neural plasticity, and rehabilitation. The ability of the brain to adapt to sensory inputs provides a powerful foundation for therapeutic interventions aimed at restoring sensory function. By leveraging the principles of cross-modal plasticity (i.e., multisensory cells activation and technological advances), innovative rehabilitation strategies can optimize recovery outcomes.

The integration of multisensory-based training programs with novel therapeutic tools such as optogenetics and virtual reality presents a promising frontier in clinical applications. These approaches not only enhance sensory compensation but also pave the way for breakthroughs in restoring functional vision in visually impaired individuals. Future research should continue refining these methods to maximize their effectiveness, ensuring that multisensory rehabilitation remains at the forefront of neuroscience-driven medical advancements.
